# Influence of Metal of the Applicator on the Dose Distribution during Brachytherapy

**DOI:** 10.1371/journal.pone.0104831

**Published:** 2014-08-18

**Authors:** Chin-Hui Wu, An-Cheng Shiau, Yi-Jen Liao, Hsin-Yu Lin, Yen-Wan Hsueh Liu, Shih-Ming Hsu

**Affiliations:** 1 Institute of Nuclear Engineering and Science, National Tsing Hua University, Hsinchu, Taiwan, ROC; 2 Department of Biomedical Imaging and Radiological Sciences, National Yang-Ming University, Taipei, Taiwan, ROC; 3 Department of Radiation Oncology, Koo Foundation Sun Yat-Sen Cancer Center, Taipei, Taiwan, ROC; 4 School of Medical Laboratory Science and Biotechnology, Taipei Medical University, Taipei, Taiwan, ROC; 5 Department of Radiation Oncology, Saint Mary's Hospital Luodong, Yilan, Taiwan, ROC; 6 Biophotonics and Molecular Imaging Research Center, National Yang-Ming University, Taipei, Taiwan, ROC; Northwestern University Feinberg School of Medicine, United States of America

## Abstract

This study explores how the metal materials of the applicator influence the dose distribution when performing brachytherapy for cervical cancer. A pinpoint ionization chamber, Monte Carlo code MCNPX, and treatment planning system are used to evaluate the dose distribution for a single Ir-192 source positioned in the tandem and ovoid. For dose distribution in water with the presence of the tandem, differences among measurement, MCNPX calculation and treatment planning system results are <5%. For dose distribution in water with the presence of the ovoid, the MCNPX result agrees with the measurement. But the doses calculated from treatment planning system are overestimated by up to a factor of 4. This is due to the shielding effect of the metal materials in the applicator not being considered in the treatment planning system. This result suggests that the treatment planning system should take into account corrections for the metal materials of the applicator in order to improve the accuracy of the radiation dose delivered.

## Introduction

Statistics from the World Health Organization indicates that cervical cancer is the second most common cancer among women after breast cancer [1]. In addition to invasive surgical procedures, the main treatments for cervical cancer now include chemotherapy, external-beam radiotherapy, and brachytherapy [2], [3].

The 5-year survival rates of patients with Stage IB or IIA cervical cancer who receive external-beam radiotherapy together with brachytherapy are about 80∼90%, which is as high as for patients treated with hysterectomy together with pelvic lymph-node dissection [4]. Moreover, radiation therapy is better than surgical treatment for controlling the tumor in patients with later stages of cervical cancer [5]. Brachytherapy delivers a radiation dose to the tumor over a short distance. The steep dose gradient result in the tumor receiving a high dose within a short time and reduces the doses received by adjacent healthy tissues. Brachytherapy doses are currently calculated based on the TG-43 report of The American Association of Physicists in Medicine [6]. The parameters and dose calculation formulas provided in that report are based on the assumption of a pure water environment. However, the change in the brachytherapy dose distribution in actual treatments due to the inhomogeneous media of the human body is an important issue clinically [7].

An applicator is used to position the radioactive source within the patient's body when performing brachytherapy treatment for cervical cancer. Usually the applicator has a metal material covering its outside and might even also contain a metal structure within the ovoid. Henschke applicators have been widely used inpatients with cervical cancer in recent years [8]–[11], but the dose perturbation caused by such applicators is not currently evaluated in treatment planning system. The European Society for Therapeutic Radiology and Oncology(ESTRO) suggested considering the effects of tissue inhomogeneity and source shielding during treatment planning system [12]. Watanabe used thermoluminescent dosimeter and MCNP4Acode to perform dose measurements and dose calculations for a Henschke applicator, with the results revealing uncertainty between the MCNP4A-calculated value and the actual dose measured [13].

The present study aims to determine how the dose distribution is influenced by shielding materials contained in the ovoid foranIr-192 source inside the Henschke applicator. Monte-Carlo N-Particle eXtended (MCNPX) code used in this study to simulate the dose distribution of the Henschke applicator, and a small ionization chamber is used for measurements. The measured values are compared with the results of MCNPX and the results of the treatment planning system. The deviation of dose delivery caused by dosimeter displacement is also explored.

## Materials and Methods

### Ir-192 source and Henschke applicator

**Figure 1:**
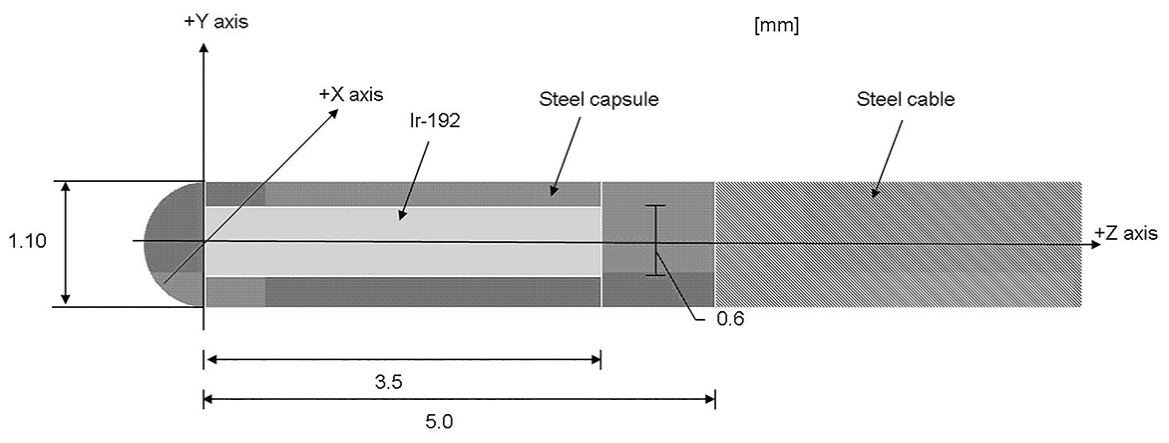
Structural diagram of the Ir-192 source and its stainless steel outer cover.

**Figure 2:**
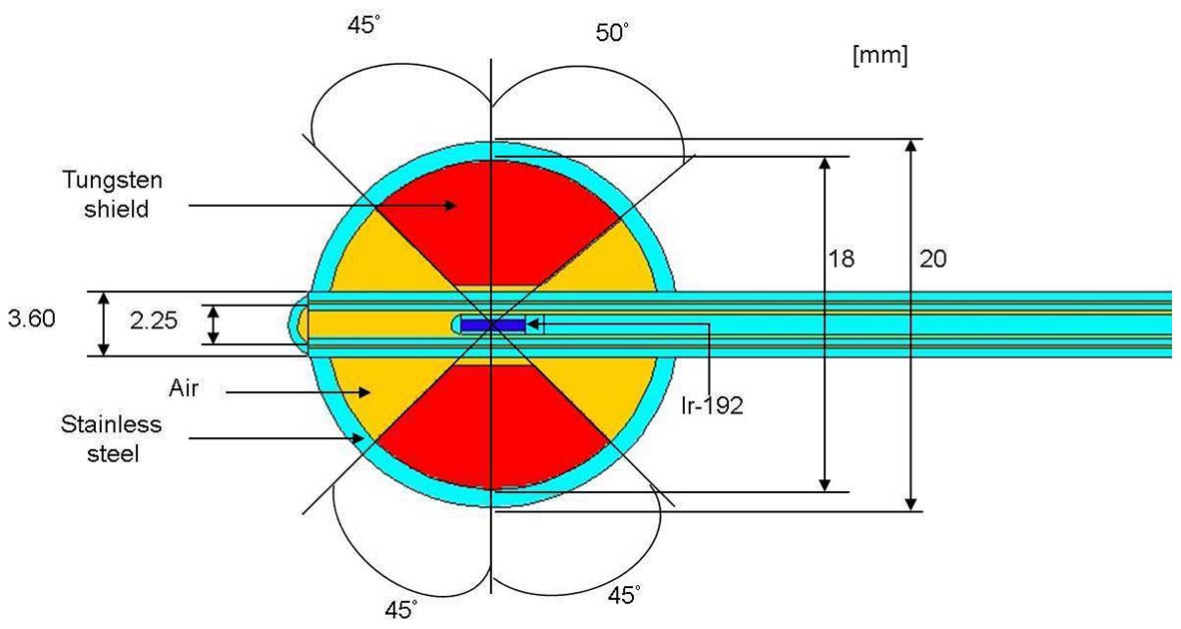
Structural model of Henschke applicator for Ir-192 source for MCNP calculation, visualized by MCNPX Visual Editor.

The Ir-192 source used in this study is 3.5 mm long with a diameter of 0.6 mm. It is covered by stainless steel with an outer diameter of 1.1 mm. The source is attached to a remote after-loading machine (MicroSelectron HDR, Nucletron, Netherlands) by a stainless steel wire. Fig. 1 shows the geometric diagram of the source. Fig. 2 shows the Henschke applicator, which comprises of a tandem and ovoid son its two sides. The applicator functions as the translation pathway for the source during cervical cancer treatment. The ovoid contains tungsten alloy as a shielding material with a density of 17.0 g/cm^3^, the composition of which is 91% tungsten, 4.5% nickel, and 4.5% iron. Kodak X-Omat V films are used to capture the shielding structure and layout inside the ovoid of the Henschke applicator. MCNPX code is used to simulate the Henschke applicator and Ir-192 source. With the aid of MCNPX Visual Editor, the applicator model set up is shown in Fig. 2.

### Experimental setup of dose distribution measurement using Henschke applicator

In this study a water phantom (MT-100-T, MED-TEC, USA) with dimensions of 30.5 cm×38 cm×38 cm is used to perform measurements with Ir-192 located at a single position to determine how the metal materials of the Henschke applicator influences the dose distribution. This experiment used a 0.01-ccpinpoint ionization chamber (CC01, IBA Dosimetry, Germany) to measure the dose distribution for the ovoid and tandem. The X, Y, and Z axes correspond to left–right, front–back, and up–down directions, respectively. The measurement area is 0 mm in the X direction, from 15 mm to 45 mm in the Y direction, and from −40 mm to 40 mm in the Z direction.

### Dose distribution calculation using MCNPX

In this study the differences among the ionization-chamber-measured, treatment-planning, and MCNPX-calculated values are compared. The reliability of the measured values can be confirmed by MCNPX results.

### Comparison of the MCNPX F6 and ^*^F8 tallies

MCNPX was developed by the Los Alamos National Laboratory (LANL) [14]. Two tally methods F6 & *F8 are used to calculate the MCNPX photon dose for comparison. F6 tally is based on the assumption that electronic balances exists in the tally region, and that an electron would lose its energy instead of undergoing electron transport tat the position where a photon and electron collide. ^*^F8 tally considers the energy deposition of an electron on its migration path when a photon and electron collide, rather than the instantaneous energy deposition at the position of the collision. The ^*^F8 tally can reflect more truly the actual migration of radiation particles and provides more accurate calculation results, but the computing time is longer.

Both the F6 and ^*^F8 tallies are used to calculate the Ir-192 dose distribution in the water phantom to see the dose differences between these two tally methods. The simulation includes only the Ir-192 source, and not the Henschke applicator. The Ir-192 photon spectrum was obtained from Brookhaven National Laboratory [15]. Tally spheres with diameter of 1 mm are placed at distances of 15 mm and 25 mm on the Y axis. Tally spheres with diameter of 3 mm are placed at distances of 35 mm and 45 mm on the Y axis. For number of particles equals 5×10^8^, the statistical errors of less than 3%. In addition, the F6 tally is used to calculate anisotropy functions and radial dose functions for comparison with the results of Williamson and Li [16].

### Monte Carlo calculation for the dose distribution in water with presence of the Henschke applicator

For the dose distribution in water with presence of the Henschke applicator, MCNPX code is used to calculate the dose to clarify the reliability of the ionization-chamber-measured values. The conditions of the simulation are made as close as possible to those in actual measurements. The F6 tally is used for dose calculations in the simulation in order to reduce the calculation time and statistical errors. The MCNPX-calculated, ionization-chamber-measured, and treatment-planning calculated values are compared to assess how the metal materials influence the dose distribution.

### Dose variation caused by imprecision of measurement position

**Figure 3:**
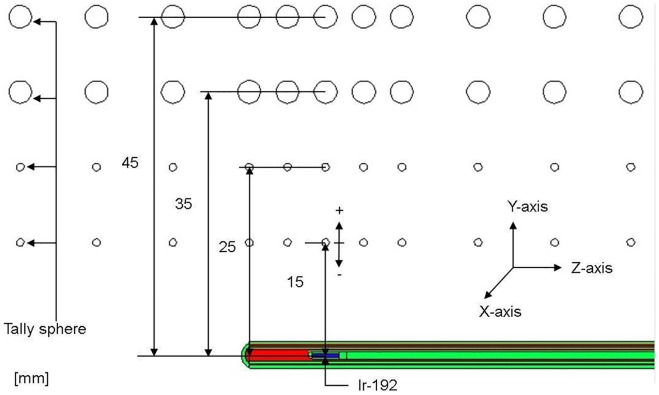
Diagram of the setup for simulating dose variation caused by imprecision of measurement-position MCNPX tally spheres are placed at different distances from the source. Positive/negative signs indicate positions away from or close to the source, respectively.

Brachytherapy is associated with steep dose gradients. To evaluate dose variation caused by imprecision of positioning during the experiment, MCNPX is applied to simulate the situation of an Ir-192 source at a fixed position. The tally spheres are at 15 mm, 25 mm, 35 mm, and 45 mm away from the source as shown in Fig. 3. The imprecision of measurement position are ±0.25 mm, ±0.50 mm, ±1.0 mm, and ±2.0 mm along the Y axis.

## Results and Discussion

### Differences between F6 and ^*^F8 tallies in MCNP calculation

**Figure 4:**
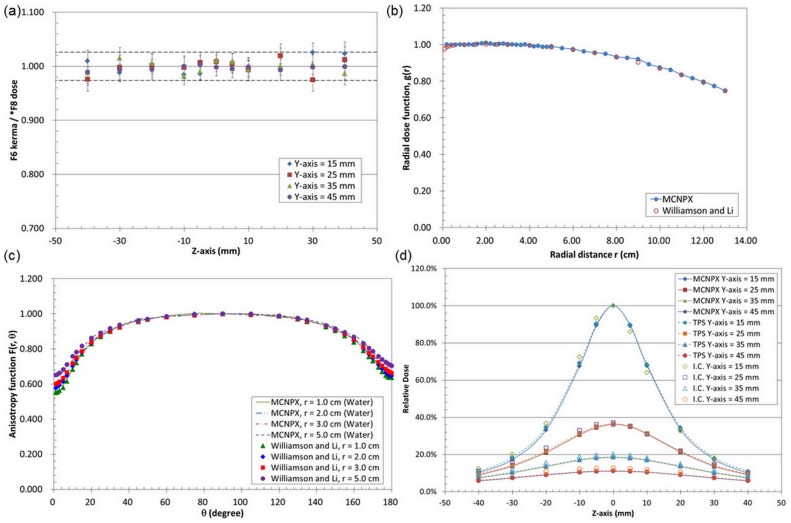
Monte Carlos simulation. (a) Ratios of F6 and ^*^F8 tally dose of MCNPX calculation for the presence of Ir-192 in the water phantom. (b) Comparison of MCNPX-calculated and previously reported results for the Ir-192 radial dose function. (c) Comparison of MCNPX-calculated and previously reported results for the Ir-192anisotropy function. (d) Comparison of dose distribution in water with the presence of the tandem for the ionization-chamber-measured value (I.C.), treatment-planning system value (TPS), and MCNPX-calculated value.

The ratio of doses calculated by using F6 and ^*^F8 tallies on the in MCNPX calculation is shown in Fig. 4(a). The value lies between 0.97 to 1.03. That is the differences between these two types of tally are small, <3%. However, the computing time of *F8 tally is ∼30 times longer than that of the F6 tally. Therefore, performing an Ir-192 dose calculation using MCNPX, F6 tally can be used when the environment media is homogeneous to save computing time.

### Radial dose function and anisotropy function

Figure 4(b) shows the radial dose function, i.e. the dose reduction profile caused by photon absorption and scattering while the photons travel along the transverse direction of the source calculated by using MCNPX. The results agree well with those of Williamson and Li [16]. The differences are <1.8%, less than the 2σ statistical error ( = 3%) of the MCNPX calculation. The number of particle used in MCNPX calculation is 5×10^8^.

The anisotropy function accounts for the anisotropy of dose distribution around the source, including the effects of absorption and scatter in the medium. The calculation results are compared with those of Williamson and Li [16] as shown in Fig. 4(c). For region of 1 cm radius the differences are within 4.6% for θ<5°, within 2.5% for 5°<θ<180°. For region outside 1 cm radius, the differences are within 2%.All are within 2σ of MCNPX calculations.

### Dose distribution in water with the presence of the tandem

To evaluate the accuracy of the measured values under the situation with presence of tandem, this study compared the measurement results with the calculated results of MCNPX and the treatment-planning system. The measured and the calculated results of dose distribution in water with the presence of the tandem are shown in Fig. 4(d). The measurement and the calculated results of treatment-planning and MCNPX are all normalized to dose at Y = 15 mm and Z = 0 mm. The MCNPX results shows that in Y-direction, the dose at 15 mm, 25 mm, 35 mm and 45 mm drop from 1 to 0.36, 0.18 and 0.11, almost follows the inverse square law. The average dose reduction is 6.4% per mm in Y direction. For Y = 15 mm, the dose along z-direction changes from 1 at z = 0 to 0.68 at z = 10 mm. The average dose reduction in Z-direction is 3.2% per mm. The dose gradient is steeper along the Y direction than along Z direction. Hence the dose errors due to imprecision location would be greater along the Y direction.

**Figure 5:**
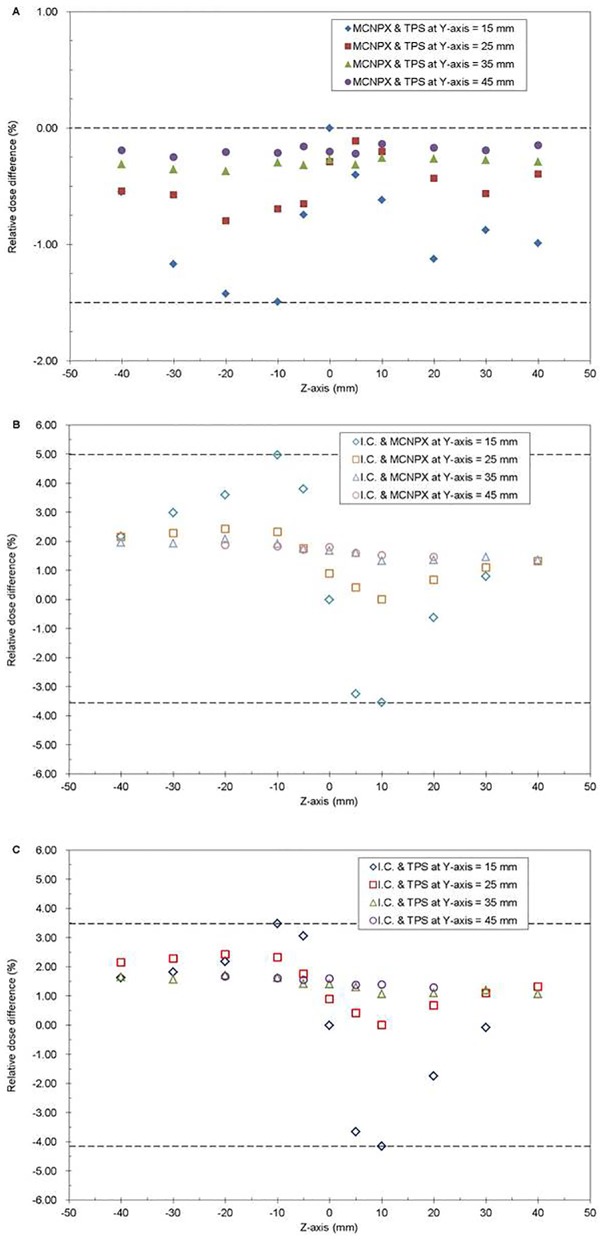
Relative dose differences in water with the presence of the tandem. (a) MCNPX-calculated value versus treatment-planning value, (b) MCNPX-calculated value versus ionization-chamber-measured value, and (c) treatment-planning value versus ionization-chamber-measured value.

The comparison of this three relative dose distribution are shown in Fig. 5. After normalized, the dose differences between the MCNPX-calculated and treatment-planning results are <1.5%, within 2σ of the Monte Carlo calculation. The difference decreases as distance from source increases. The dose differences between the ionization-chamber-measured and MCNPX-calculated values are <5%; the dose differences between the measured and the treatment-planning system values are also <5%.

### Dose distribution in water with the presence of the ovoid

**Figure 6:**
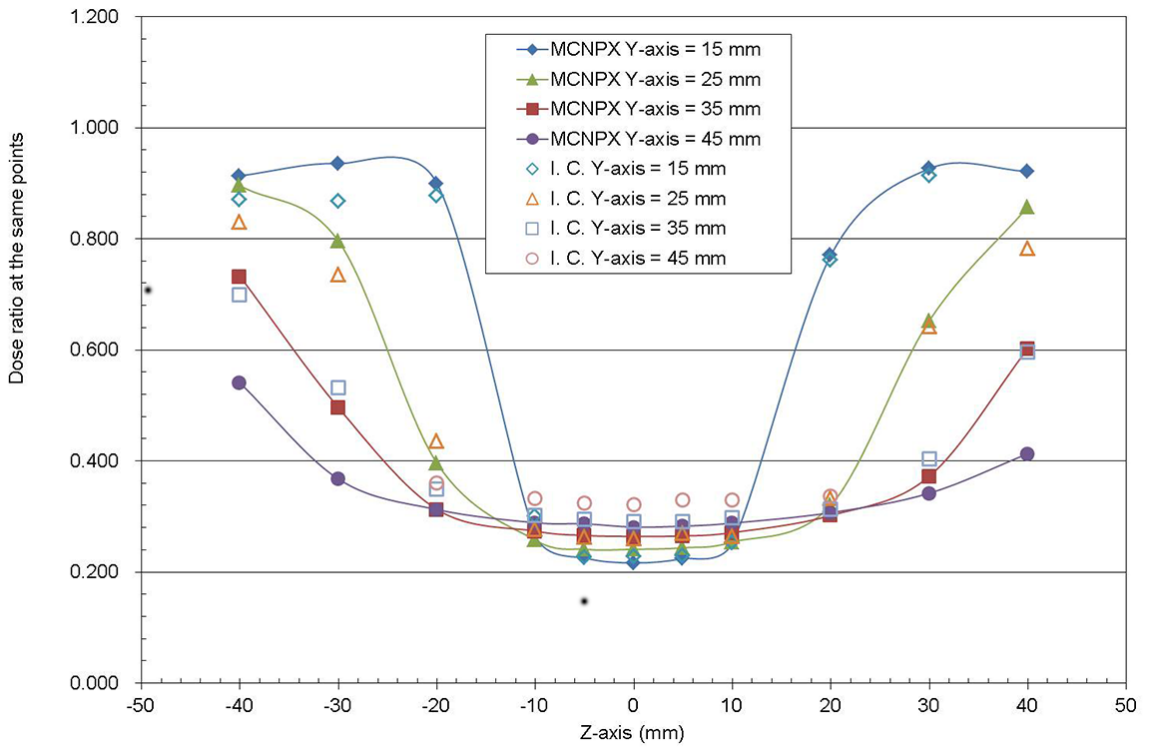
The ratio of dose with the presence of the ovoid and with the presence of the tandem.

**Figure 7:**
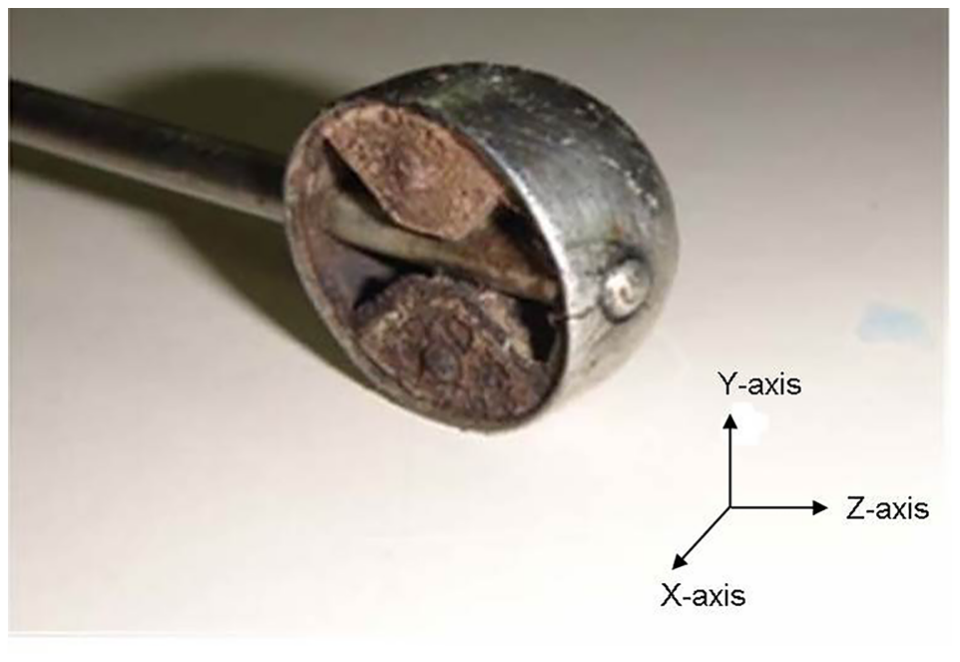
Photograph showing the internal structure of the ovoid of the Henschke applicator.

Current treatment planning system for brachytherapy does not consider the dose attenuation due to the metal inside the ovoid. The shielding effect of metal inside the ovoid is analyzed for both measured and MCNPX calculation results. The ratios of dose calculated by MCNPX with the presence of ovoid to dose with tandem present only are shown in Fig. 6. For Y = 15 mm, the ratio changes from 0.94 at outside region to 0.22 at inside region (<+−10 mm, as shown in Fig.7). The effect is less severe as the distance from the source axis increases. The difference of dose ratio between the measured and the MCNPX calculated values are ∼5–8%.Therefore, if the shielding effect of the ovoid is neglected, the dose predicted by treatment planning system can be overestimated by up to a factor of ∼4.

The bladder and rectum are located within the adjacent areas above and beneath the Y axis. It is verified by both clinical measurements and MCNPX calculations that the Henschke applicator does provide shielding effect and can reduce the doses to the bladder and rectum. The current treatment-planning system which neglects this metal-shielded area on the other hand, will overestimate the dose of the shielded area by a factor of as large as 4. The above result also implies that if the tumor is located behind the ovoid, the tumor dose will be overestimated if the shielding effect of the metal is not taken into consideration. The magnitude of the overestimation of dose depends on the dwelling time of the source in the region shielded by the metal of ovoid from the source location.

### Dose deviation caused by the imprecise positioning

The dose deviation caused by imprecise measurement-position calculated by MCNPX is shown in Table 1. The dose deviation for imprecise positioning of ±0.25 mm is small, ∼3%, comparable to the 2σstatistical error. For imprecise positioning of ±0.5 mm, ±1 mm, and ±2 mm, the dose deviation are 6∼7%, 12∼15% and 22∼33% respectively.

**Table 1:** Dose variation caused by measurement-position errors, calculated by MCNPX (1σ<1.5%).

	Dose variation (%) with change of the vertical distance (Y-axis)									Dose variation (%) with change of the vertical distance (Y-axis)							
	Distance error = −0.25 mm				Distance error = +0.25 mm					Distance error = −1.00 mm				Distance error = +1.00 mm			
Z-axis (mm)	15 mm distance	25 mm distance	35 mm distance	45 mm distance	15 mm distance	25 mm distance	35 mm distance	45 mm distance	Z-axis (mm)	15 mm distance	25 mm distance	35 mm distance	45 mm distance	15 mm distance	25 mm distance	35 mm distance	45 mm distance
40	−0.10	0.64	0.57	0.71	−0.04	−0.34	−0.51	−0.59	40	−0.32	2.69	2.47	2.71	0.26	−1.93	−2.00	−2.86
30	−0.42	1.31	0.84	0.75	−0.82	−0.24	−0.78	−0.86	30	0.20	2.80	3.00	3.34	−0.56	−3.02	−3.12	−2.93
20	0.40	1.52	1.11	0.90	−0.41	−0.93	−0.99	−0.94	20	3.53	4.38	4.37	4.10	−3.91	−4.29	−4.05	−3.57
10	2.25	1.65	1.43	1.02	−2.37	−1.71	−1.40	−1.07	10	9.51	6.89	5.69	4.33	−8.53	−7.01	−5.30	−4.11
5	3.08	1.74	1.43	1.12	−3.07	−1.72	−1.34	−1.11	5	12.97	7.43	5.90	4.62	−11.00	−6.45	−5.28	−4.26
0	3.45	2.01	1.46	1.17	−3.13	−2.01	−1.42	−1.08	0	14.81	8.25	5.91	4.74	−12.10	−7.78	−5.57	−4.40
−5	3.11	2.03	1.37	1.14	−2.91	−1.81	−1.39	−1.11	−5	12.95	8.40	5.78	4.71	−10.67	−7.07	−5.38	−4.34
−10	2.23	1.95	1.39	1.10	−1.96	−1.58	−1.33	−1.05	−10	9.84	8.10	5.58	4.50	−8.16	−6.45	−5.29	−4.23
−20	1.33	1.44	1.18	0.88	−1.63	−1.55	−1.12	−0.87	−20	4.16	5.32	4.87	3.88	−4.34	−4.93	−4.20	−3.18
−30	0.83	0.32	0.84	0.69	−0.53	−0.90	−0.86	−0.61	−30	1.42	2.05	3.17	2.90	0.53	−4.06	−3.32	−2.75
−40	−0.47	0.86	0.69	0.60	−0.03	0.11	−0.70	−0.56	−40	−1.24	2.51	2.66	2.19	−0.23	0.19	−2.61	−2.40
	Distance error = −0.5 mm				Distance error = +0.5 mm					Distance error = −2.00 mm				Distance error = +2.00 mm			
Z-axis (mm)	15 mm distance	25 mm distance	35 mm distance	45 mm distance	15 mm distance	25 mm distance	35 mm distance	45 mm distance	Z-axis (mm)	15 mm distance	25 mm distance	35 mm distance	45 mm distance	15 mm distance	25 mm distance	35 mm distance	45 mm distance
40	−0.42	1.95	1.17	1.40	0.36	−0.73	−0.99	−1.34	40	−1.15	4.39	5.11	4.64	−0.62	−2.40	−4.00	−5.10
30	−0.33	2.21	1.53	1.73	−0.78	−1.00	−1.52	−1.59	30	2.10	6.50	6.42	6.29	−3.49	−7.79	−6.19	−5.89
20	1.03	3.21	2.21	1.95	−1.30	−2.11	−2.00	−1.78	20	8.57	8.88	8.96	8.49	−8.14	−10.47	−7.71	−6.90
10	4.74	3.35	2.81	2.05	−4.54	−3.53	−2.74	−2.02	10	19.87	14.38	11.64	9.06	−15.86	−13.68	−10.14	−7.97
5	6.28	3.48	2.88	2.30	−5.84	−3.25	−2.67	−2.18	5	28.59	16.34	12.37	9.78	−20.47	−13.35	−10.38	−8.31
0	7.07	3.93	2.93	2.32	−6.24	−3.98	−2.83	−2.21	0	33.00	18.00	12.53	9.91	−22.04	−14.57	−10.65	−8.60
−5	6.38	4.18	2.81	2.33	−5.70	−3.44	−2.73	−2.17	−5	28.69	17.30	12.28	9.36	−19.94	−13.89	−10.48	−8.43
−10	4.73	3.91	2.75	2.19	−4.05	−3.04	−2.69	−2.17	−10	20.04	16.67	11.59	9.04	−15.93	−12.90	−10.45	−8.25
−20	2.30	2.68	2.42	1.88	−2.37	−2.70	−2.12	−1.60	−20	8.47	10.79	9.30	8.00	−8.05	−7.74	−8.03	−6.50
−30	0.96	0.74	1.61	1.36	−0.11	−1.93	−1.71	−1.40	−30	2.92	5.70	6.50	6.52	−2.37	−6.42	−6.66	−5.35
−40	0.38	1.95	1.32	1.11	−0.42	0.80	−1.39	−1.16	−40	0.35	4.30	4.96	4.31	−3.15	−1.78	−4.83	−5.02

The dose deviation caused by imprecise positioning tended to decrease with distance from the source. Special attention should therefore be paid to precise positioning when measuring doses in areas near the source location. This observation also implies that if the source location relative to tumor is imprecise, dose error up to 17% per mm would occur. Therefore, the applicator setup errors should be reduced to within 0.25 mm to improve the accuracy of the dose delivery during the brachytherapy.

## Conclusions

This study used a pinpoint ionization chamber to perform dose measurements for the Henschke applicator containing a source at a single point. In addiation to the treatment planning system, MCNPX is used to simulate the experimental condition to confirm the measurements. For dose calculation with presence of tandem, the three results, after normalized, agree well with one another.

For dose calculation with presence of ovoid, the MCNPX-calculated and ionization-chamber-measured values agree well <5%, the difference is within 2σ of MCNPX calculation.

The treatment planning system, which currently does not take into account the shielding material in ovoid, overestimation of the dose by up to a factor of 4 is possible. This results show that the shielding material inside the ovoid of the Henschke applicator can reduce the dose delivered to adjacent healthy organs. However, the tumor located behind the ovoid, its dose will also be overestimated by the treatment planning calculation. Corrections should be made for the presence of the Henschke applicator to ensure the accuracy of the doses delivered.
